# Movements Execution in Amnestic Mild Cognitive Impairment and Alzheimer’s Disease

**DOI:** 10.1155/2007/845914

**Published:** 2007-08-22

**Authors:** Rosolino Camarda, Cecilia Camarda, Roberto Monastero, Silvia Grimaldi, Lawrence K. C. Camarda, Carmela Pipia, Carlo Caltagirone, Massimo Gangitano

**Affiliations:** ^1^Laboratory of Epidemiology and Psychology of Aging and DementiaSection of NeurologyDepartment of Clinical NeuroscienceUniversity of PalermoPalermoItaly; ^2^Department of NeurologyUniversity "Tor Vergata"and Fondazione "Santa Lucia" IRCCSRomeItaly

**Keywords:** Alzheimer’s Disease, Mild Cognitive Impairment, kinematics, pointing, neuropsychology

## Abstract

We evaluated the relationship between motor and neuropsychological deficits in subjects affected by amnestic Mild Cognitive Impairment (aMCI) and early Alzheimer’s Disease (AD). Kinematics of goal-directed movement of aMCI and AD subjects were compared to those of age-matched control subjects. AD showed a slowing down of motor performance compared to aMCI and controls. No relationships were found between motor and cognitive performances in both AD and aMCI. Our results suggest that the different motor behaviour between AD and aMCI cannot be related to memory deficits, probably reflecting the initial degeneration of parietal-frontal circuits for movement planning. The onset of motor dysfunction in early AD could represent the transition from aMCI to AD.

## 1. Introduction

Alzheimer disease (AD) is the most common form of dementia, accounting for 50% to 70% of all cases [17]. Multiple cognitive deficits and functional impairment are the main features of dementia and AD, characterizing the diagnostic criteria currently used [3,29]. However, because of worse prognosis, other symptoms such neuropsychiatric abnormalities [4] and motor impairment [14] have to be considered. AD pathology exists covertly over a period of months to years before the onset of clinically detectable symptoms [11]; the early recognition of subtle cognitive impairment due to the disease from cognitive changes of normal aging is sometimes not easy.

In the last years, the status of subtle cognitive impairment in nondemented elderly has attracted intense interest. Within the different syndromes with variable prognosis proposed [13] the construct of Mild Cognitive Impairment (MCI) [39] has been one of the most commonly investigated. According to Petersen et al. [39] original definition, the term MCI refers to individuals with subjective memory complaint, impaired memory testing, normal general cognitive functioning and relatively intact activities of daily living. These criteria basically centered on impaired memory, the so-called amnestic Mild Cognitive Impairment (aMCI), have been broadened recently to encompass other non-memory cognitive domains with a subsequent description of other MCI subtypes [40]. Subjects with aMCI show an high rate of progression to AD with an annual conversion rate of about 12% vs 1–2% in cognitively intact elderly [39].

As well as for cognitive functioning, a continuum regarding age-related changes and disability due to Alzheimer's disease may be hypothesized also for motor functions since abnormalities in these functions increase in frequency and severity over time coupling the ongoing decline of cognitive functions [46,50]. The clinical examination of AD patients often shows sever-

ISSN 0953-4180/07/$17.00 © 2007 – IOS Press and the authors. All rights reserved al types of extrapyramidal signs (EPS) [46,50]. EPS in AD increase in prevalence as the disease advances [46, 50] and are associated with cognitive and functional decline [46,50], institutionalization [46], increased costs of care [32] and death [46]. EPS in AD are associated with substantia nigra pathology although causative lesions for EPS outside the extrapyramidal system cannot be excluded [8].

Recent population-based cohort studies showed that motor function is impaired in subjects with MCI [1, 27] and that the degree of EPS in lower extremity is related to the risk of AD [1]. By contrast, in a clinical-based study, the assessment of motor function with performance-based tests failed to show any impairment in MCI subjects [42].

Very few studies to date have applied quantitative instrumental methods to evaluate motor functions in cognitively impaired elderly subjects [20,21,23,47]. Using these methods, slight motor dysfunctions have been found in the early stage of AD [20,21,23], probably accounting for memory impairment. These data were recently confirmed by a German kinematic study conducted in aMCI subjects, who showed loss of fine motor performance compared to healthy controls [47]. However, in this article the authors did not controlled for coexistent depression and extrapyramidal motor signs, all factors that may have interfered with motor performances.

According to previous findings it is not clear whether in cognitively impaired elderly motor failure matches the decline of memory function or alternatively is coupled with the decline of other cognitive functions than memory. To address this issue, using a visuomotor integration task, we studied the kinematics of the upper limb of a group of subjects with mild-to-moderate AD and compared them with the kinematics of a group of subjects with aMCI. Motor performance of both groups were then related to neuropsychological scores obtained in cognitive tasks. To control for confounding we excluded subjects with mild to severe EPS, depressive symptoms or under psychotropic medications.

## 2. Materials and methods

### 2.1. Subjects

Eleven patients with AD (6 women and 5 men), 11 with aMCI (6 women and 5 men) and 11 healthy controls (6 women and 5 men) participated to the study. Diagnostic evaluation of AD and aMCI patients included physical and neurological evaluation, neuropsychological testing, laboratory testing, and neuroimaging with brain computed tomography or magnetic resonance imaging.

The diagnosis of dementia was based on the Diagnostic and Statistical Manual of Mental Disorders, $4^{th}$ edition (DSM-IV-TR) [3], and the diagnosis of AD was based on the criteria of the National Institute for Neurological and Communicative Disorders and Stroke-Alzheimer Disease and Related Disorders Association (NINCDS-ADRDA) [29]. Only mild-to-moderate AD subjects were included (i.e. Mini-Mental State Examination [MMSE] [16] scores ranged from 17 to 24).

Subjects with aMCI were ascertained according to the criteria proposed by Petersen et al. [39], including: (a) memory complaints reported by the subject or the informant (or both); (b) objective memory impairment for age and education (1.5 SD below the age- and education adjusted scores); (c) normal global cognitive function (MMSE score $> 24$); (d) intact activities of daily living (Activities of Daily Living [ADL] score $=$ 6/6) [26]; (e) Clinical Dementia Rating (CDR) score of 0.5 [31]; (f) absence of dementia (DSM-IV-TR criteria) [3]. Neither aMCI nor AD subjects were on psychotropic medications (i.e. neuroleptics, anxyolitics including benzodiazepines other tranquilizers and hypnotic, and antidepressants) or experienced depressive symptoms (i.e. Cornell Depression Scale score $\leqslant 7$) [2].

Controls were matched for gender, age and education to aMCI and AD subjects. Inclusion criteria were: (a) no diagnosis of major medical, neurological or psychiatric illness; (b) no history of significant head injury or substance abuse; (c) no evidence of dementia, functional (ADL $=$ 6/6) or cognitive decline (MMSE $> 27$) on examination.

For all subjects common inclusion criteria were: (a) the absence of any kind of tremor in upper extremities and the presence of mild-to-absent EPS (i.e. a score $<$ 7 at the Motor Examination of the Unified Parkinson's Disease Rating Scale [UPDRS-ME] [15]); (b) being right-handed according to the Edinburgh Inventory of handedness [35].

All subjects gave their informed consent prior to their inclusion to the study, which was approved by the local Ethical Board.

### 2.2. Neuropsychological evaluation

The neuropsychological battery included tests of verbal long-term memory (Rey's Word List Learning

Immediate and 10 min Delayed Recall) [10], verbal and spatial short-term memory (Digit Span and Corsi Span) [36], constructional abilities (Copy Drawings with and without Landmarks) [18], attention and psychomotor speed (Attentive Matrices and Trial Making Test part A, TMT-A) [22,49], non-verbal reasoning (Raven Coloured Matrices) [6], auditory language comprehension (Token Test) [12] and executive functions (Verbal Fluency test with phonemic cues) [34]. Tests were administered by an experienced examiner blind to results of the kinematic session and scored according to the age- and education published procedures for the Italian population [6,10,12,18,22,34,36,49].

### 2.3. Kinematic evaluation

A visuomotor integration task was executed in a separate session, run at least one day apart from the neuropsychological testing. Subjects sat in front of a table placing their right index finger on the starting position (SP) located on the table's plane, 7 cm from its edge. Six flat LEDs were placed on the table's surface that was inclined 10 degrees on the horizontal plane. LEDs were located at two distances from SP (15 and 30 cm) along three axes (midsagittal, right and left directions, oriented $0^\circ$, $60^\circ$ to the right and $60^\circ$ to the left with respect to the subject's midline, respectively). Subjects were required to point and touch with the right index finger the LED that was turned on, with their maximal velocity and accuracy. Order of target presentation was randomly assigned by the computer controlling the apparatus and the device for the motion analysis. Position of final target was varied in order to avoid any habituation effect. Movements were recorded by using the ELITE optoelectronic system (BTS, Milan, Italy) and consisted of two TV-cameras detecting infrared, reflecting passive markers. Images were acquired at the sampling rate of 100 Hz. In this study, two markers were used. The first one was placed on the nail of the index finger, the second one was placed on the table and was considered as a reference point. Subjects performed 42 trials in a total, that is 7 pointing movements for each one of the 6 possible targets.

### 2.4. Statistical analysis

Neuropsychological performance of AD and aMCI subjects was compared with two-tailed $t$-test. Concerning the kinematic evaluation, for each group of subjects (AD, aMCI and controls) mean values of reaction times (RT) movement times (MT), peaks of acceleration (PKA) peaks of velocity (PKV) and peaks of deceleration (PKD) of pointing movements were collected and analyzed. RT was the time between the switch-on of the LED on the target and the beginning of the movement; MT was considered as the interval between the beginning and the end of the movement. PKA, PKV and PKD were considered as the maximal values tangential velocity, acceleration and deceleration, respectively. PKA and PKV were chosen to evaluate the initial phase of the movement, MT was chosen to evaluate the whole movement course, PKD to evaluate the final corrective phase. Data were averaged across movement direction and target position and compared by means of ANOVA. The Newman-Keuls test was employed as post-hoc test. Neuropsychological performance in aMCI and AD was related to mean kinematics (RT, MT, PKV PKA and PKD) by means of simple linear correlation (Pearson r). For all analyses level of significance was set at $p \leqslant 0.05$.

## 3. Results

### 3.1. Demographics and clinical characteristics

Demographics of subjects are summarized in Table 1. AD and aMCI subjects were significantly different from controls on the UPDRS-ME, while do not differing each other ($p = 0.6$). As expected AD subjects showed the worst MMSE score and those with MCI performed at intermediate level between AD and controls.

### 3.2. Neuropsychological performance

Table 2 shows the mean scores of the neuropsychological tests and the corresponding p-values of pair comparisons between aMCI and AD subjects. The latter performed significantly worse than aMCI subjects in tasks evaluating short-term visuospatial memory, visuoconstructive abilities, non-verbal reasoning, attention and psychomotor speed, auditory language comprehension and executive functioning. AD and MCI subjects showed similar performance in test exploring short- and long-term verbal memory.

Table 1
Mean demographics (± SD), MMSE and UPDRS-ME scores for subjects with Alzheimer's disease, amnestic mild cognitive impairment and controls

| | AD | aMCI | Controls |
|---|---|---|---|
| Age, years | 70.82 ± 6.37 | 69.73 ± 7.88 | 69.72 ± 9.10 |
| Education, years | 6.45 ± 4.55 | 10.64 ± 5.22 | 9.73 ± 5.35 |
| UPDRS-ME | 4.27 ± 2.00* | 3.82 ± 2.32* | 1.18 ± 1.25 |
| MMSE | 20.27 ± 2.65* | 26.18 ± 1.17* | 28.45 ± 1.13 |

Abbreviations: AD = Alzheimer's disease; aMCI = amnestic mild cognitive impairment; UPDRS-ME = Motor Examination of the Unified Parkinson Disease Rating Scale; MMSE = Mini Mental State Examination.
*$p \leqslant 0.05$ vs controls.

Table 2
Mean neuropsychological scores (± SD) of subjects with Alzheimer's disease and amnestic mild cognitive impairment

| Task* | AD | aMCI | $p$ | $t$ |
|---|---|---|---|---|
| *Long-term memory* | | | | |
| Rey's Word List Learning IR | 18.4 ± 7.5 | 23.1 ± 5.8 | ns | 1.60 |
| Rey's Word List Learning DR | 2.5 ± 1.8 | 2.3 ± 1.3 | ns | 0.40 |
| *Short-term memory* | | | | |
| Corsi Span | 3.6 ± 0.9 | 4.4 ± 0.7 | 0.05 | 2.10 |
| Digit Span | 4.5 ± 1.0 | 5.1 ± 1.0 | ns | 1.22 |
| *Visuoconstructive Abilities* | | | | |
| CD with landmarks | 6.7 ± 2.3 | 8.8 ± 1.8 | ns | 1.57 |
| CD without landmarks | 52.8 ± 11.4 | 59.4 ± 7.9 | 0.03 | 2.37 |
| *Non-verbal Reasoning* | | | | |
| Raven Coloured Matrices | 15.2 ± 4.3 | 23.1 ± 7.0 | 0.001 | 3.40 |
| *Attention/Psychomotor Speed* | | | | |
| Attentive Matrices | 28.4 ± 7.5 | 39.0 ± 6.9 | 0.001 | 3.40 |
| Trail Making A | 249.5 ± 106.5 | 119.0 ± 33.5 | 0.001 | 3.88 |
| *Language* | | | | |
| Token Test | 24.50 ± 5.2 | 31.4 ± 2.5 | 0.001 | 3.89 |
| *Executive functions* | | | | |
| Phonemic Fluency | 12.27 ± 7.78 | 24.0 ± 9.22 | 0.004 | 3.22 |

*All scores are expressed as raw scores. Abbreviations: Abbreviations: AD = Alzheimer's disease; aMCI = amnestic mild cognitive impairment; IR = immediate recall; DR = delayed recall; CD = copy drawings.

### 3.3. Kinematic evaluation

Mean values of kinematics are reported in Table 3. At ANOVA significant was the difference in RT among the three groups of subjects ($F(2,30) = 5.44,\ p = 0.01$). Figure 1 depicts the differences in mean MT between the three groups of subjects ($F(2,30) = 18.90,\ p = 0.00005$). Post-hoc evaluation showed that AD were significantly different from aMCI and controls ($p < 0.0001$) whereas no differences were found between aMCI and controls. As well as for MT, same differences were found for PKA ($F(2,30) = 7.18,\ p = 0.003$), PKV ($F(2,30) = 6.81,\ p = 0.003$) and PKD ($F(2,30) = 6.61,\ p = 0.004$). Post-hoc evaluation showed again no differences between aMCI and controls and a significant reduction of maximal velocity and acceleration peaks in AD respect to the two other groups. Only for MT significant was the 3-way interaction between groups of subjects, distances and direction of movements ($F(4,60) = 4.19,\ p = 0.004$).

### 3.4. Kinematics and neuropsychological tests correlations

There was no consistent relationship between kinematics and neuropsychological scores both in AD and aMCI (data not shown).

## 4. Discussion

Few studies to date have evaluated whether a decline of motor functions in AD is related to a memory decline or, in alternative, to the involvement of cognitive

Table 3
Mean reaction times and kinematics (± SD) of subjects with Alzheimer's disease, amnestic mild cognitive impairment and controls for each distance and position of the employed targets

| | Near target | | | Far target | | |
|---|---|---|---|---|---|---|
| | Right | Central | Left | Right | Central | Left |
| *AD* | | | | | | |
| RT (ms) | 740.0 ± 32.8 | 775.3 ± 44.5 | 761.9 ± 42.5 | 815.7 ± 52.5 | 861.8 ± 64.4 | 859.8 ± 84.9 |
| PKA (mm/s$^2$) | 7477.1 ± 849.2 | 8119.5 ± 937.1 | 7039.4 ± 690.3 | 10341.5 ± 1258.7 | 9466.8 ± 1251.4 | 8616.0 ± 1069.5 |
| PKV (mm/s) | 705.2 ± 34.1 | 593.1 ± 40.6 | 509.6 ± 41.3 | 1193.5 ± 74.8 | 864.8 ± 53.6 | 759.9 ± 43.8 |
| PKD (mm/s$^2$) | 6422.6 ± 635.8 | 5179.0 ± 716.6 | 4924.1 ± 671.2 | 8561.2 ± 937.5 | 5409.7 ± 642.1 | 4622.2 ± 544.0 |
| MT (ms) | 560.1 ± 21.6 | 618.8 ± 26.4 | 660.9 ± 22.0 | 693.0 ± 32.8 | 794.3 ± 44.5 | 876.6 ± 40.2 |
| *aMCI* | | | | | | |
| RT (ms) | 599.0 ± 45.6 | 592.4 ± 47.3 | 623.5 ± 47.8 | 760.6 ± 94.2 | 751.1 ± 82.0 | 708.1 ± 89.4 |
| PKA (mm/s$^2$) | 17075.4 ± 2274.3 | 15798.0 ± 2462.8 | 12405.4 ± 1695.1 | 20867.0 ± 2849.4 | 20065.1 ± 2868.7 | 16074.1 ± 2514.5 |
| PKV (mm/s) | 1024.1 ± 89.2 | 809.4 ± 64.2 | 706.8 ± 52.9 | 1550.5 ± 141.9 | 1180.7 ± 90.4 | 1047.0 ± 73.8 |
| PKD (mm/s$^2$) | 12127.6 ± 1803.6 | 8744.1 ± 1008.0 | 8005.4 ± 1023.1 | 15170.1 ± 2022.1 | 9417.1 ± 1276.6 | 8127.8 ± 876.2 |
| MT (ms) | 430.6 ± 22.9 | 485.2 ± 22.1 | 545.6 ± 23.5 | 572.9 ± 19.4 | 617.2 ± 33.2 | 661.6 ± 30.3 |
| *Controls* | | | | | | |
| RT (ms) | 577.5 ± 13.5 | 573.6 ± 21.7 | 617.1 ± 22.7 | 630.9 ± 27.9 | 639.5 ± 35.8 | 665.8 ± 25.9 |
| PKA (mm/s$^2$) | 13318.1 ± 1028.0 | 14026.1 ± 1849.6 | 10805.1 ± 1293.5 | 17734.3 ± 1657.1 | 17737.7 ± 2167.6 | 14932.0 ± 1705.8 |
| PKV (mm/s) | 931.7 ± 40.8 | 775.3 ± 55.5 | 674.7 ± 35.8 | 1434.2 ± 49.7 | 1170.2 ± 51.1 | 1028.4 ± 33.8 |
| PKD (mm/s$^2$) | 9689.1 ± 945.9 | 8381.2 ± 877.5 | 7762.9 ± 706.3 | 11206.2 ± 882.5 | 9085.1 ± 882.2 | 8184.6 ± 669.1 |
| MT (ms) | 424.3 ± 9.1 | 460.2 ± 11.8 | 496.2 ± 11.4 | 531.5 ± 14.0 | 584.4 ± 17.3 | 635.4 ± 15.2 |

Abbreviations: AD = Alzheimer's disease; aMCI = amnestic mild cognitive impairment; RT = Reaction Time; PKA = Peak of Acceleration; PKV = Peak of Velocity; PKD = Peak of Deceleration; MT = Movement Time.

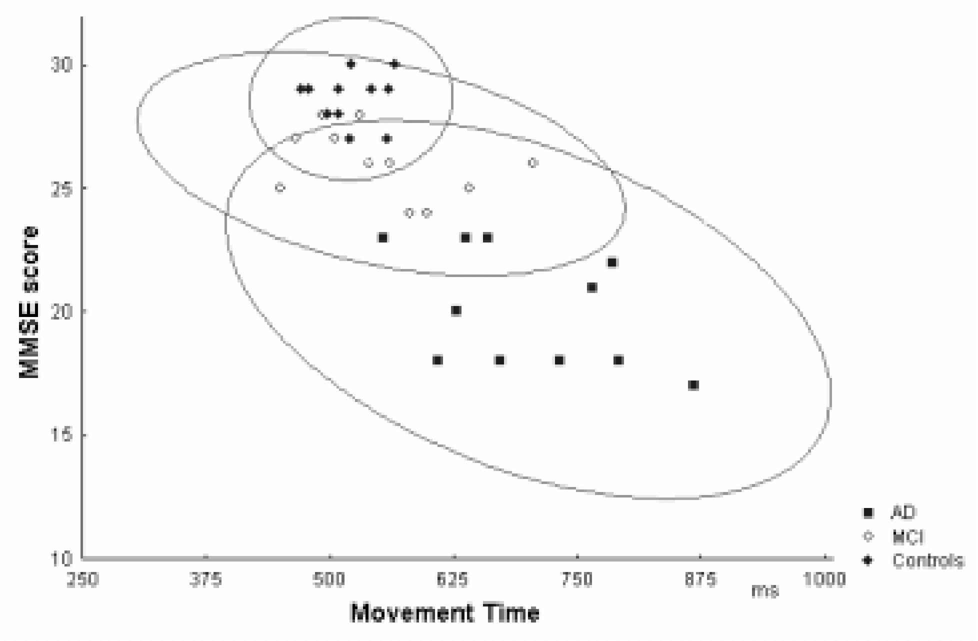

Fig. 1. Mean values of movement time (MT) of pointing movements for subjects with Alzheimer's disease (AD) (filled squares, ■), amnestic mild cognitive impairment (aMCI) (empty circles, ○) and controls (filled diamonds, ♦). For each subject data are plotted against the respective Mini-Mental State Examination (MMSE) raw score. Note the wider interval of distribution of MT of subjects with AD respect to aMCI and controls.

domains other than memory [20,21]. Furthermore, only one study has evaluated motor performance in non-demented subjects with cognitive impairment by using quantitative instrumental methods such as kinematic analyses [47]. The aim of our study was to evaluate motor performance in cognitively impaired subjects (i.e. aMCI and early AD) and to relate motor function of these subjects to cognitive performance in memory and nonmemory tasks.

The main result that emerges from our study is the presence of a slight, not significant, motor dysfunction in aMCI subjects and the presence of a remarkable slowing down of pointing in AD subjects. This slowing down was distributed throughout the whole movement time course affecting both the initial programmative phase (indexed by RT and PKA) and the final part of movement when corrective adjustments were made (indexed by PKD). These data may not be accounted for the presence of EPS since we include in the analyses only subjects with mild-to-absent EPS, and differences in UPDRS-ME scores between AD and aMCI were negligibile. Furthermore, our results cannot be explainable in terms of motor slowness due to coexistent depression or psychotropic medications, the latter being associated with EPS even when administered in very low doses [9], because at the enrollment we have excluded subjects with depressive symptoms or under psychotropic treatment.

It is widely accepted that kinematics of goal-directed movements reflect the features of visuomotor transformation processes for motor planning [5,19,30]. In AD such kind of movements is disrupted being characterized by slowing down and inaccuracy of execution [7, 20,21]. Previous studies have shown that such features are due to the presence of multiple re-accelerations that segment the whole movement both in the accelerative and in the decelerative phases [7,20,21]. These abnormalities have been associated with the severity of memory decline per se and secondarily with the involvement of other cognitive domains [20,21]. Recently, this hypothesis was confirmed in aMCI subjects by a kinematic study of handwriting conducted by Schr öter et al. [47]. Indeed, the authors reported that a loss of fine motor performance can be found in both AD and aMCI subjects, regardless the amount of memory decline. On the opposite, others have shown that AD but not aMCI subjects have impaired motor function [42]. Our data support these findings. In fact, since in our sample aMCI subjects were not distinguishable from controls in the clinical evaluation of motor function (i.e. UPDRS-ME), but shared with mild AD comparable memory deficits, the compromising of motor performance in mild AD may be only accounted for the comprehensive impairment of other non-memory cognitive domains. Different results between our and those described by Schröter et al. [47] could be accounted for, as suggested by the same authors, the presence of depressive symptoms commonly observed in subjects with MCI [28] or coexistent EPS not controlled in their study.

We hypothesize that the motor impairment showed by mild AD subjects probably reflects the compromising of parietal-frontal circuits for motor programming [23]. Planning of aimed reaching movements requires the transformation of spatial targets properties (i.e. position) with respect to the body of the subjects executing the movement [25]. Visual and proprioceptive-kinestetic informations are transformed in a series of commands integrated in specialized circuits located in the posterior parietal cortex [25]. The execution of a goal-directed movement depends not only on the efficiency of planning but also on the efficient evaluation of spatial properties of targets and consequently on the proper allocation of spatial attention [37].

It is well known that in AD the loss of memory is linked to the early involvement of medial temporal and hippocampal cortices whereas other cortices are compromised only in successive phases of the disease [41]. On the other hand, metabolic studies in subjects with AD have shown an early pathological hypoperfusion of the posterior parietal regions feeding the premotor areas [24,33]; these are the same areas that are concerned with visuomotor transformation for planning of aimed movements. According to these studies, the quasi-to-normal motor performance observed in our aMCI sample could be due to the preserved integrity of parieto-frontal circuits.

In our task, subjects had to choose a position among six possible targets. Every target was visible from the beginning and was a potential goal for the pointing, thus they had to look at the entire apparatus in order to identify the target that they have to point to. This was probably obtained shifting their attention from a primary, neutral position toward the actual target (i.e. the led to switch-on). In other words, task required an initial recruitment of attentive resources. The capability to properly select motor targets, still preserved in healthy elderly people [48] seems to be compromised in subjects with AD probably because of a defective disengagement of gaze from non-relevant visual cues in favor of the impending movement target [37,38].

However, AD were slower than aMCI not only in the initial phases (smaller PKA and PKV) but in the whole movement (longer MT). The compromising of parieto-frontal circuits could explain also the differences between AD and aMCI in the late phases of movement since parietal circuits subserve not only attentive functions [43–45], but also visuo-motor functions for motor planning and movement re-programming [25].

The strengths of this study are the strict inclusion criteria and the sophisticated kinematic approach to study motor function. However, some methodological issues deserve comment. First, the cross-sectional design of our study. Motor signs may fluctuate over time and may be not present on every examination [50]. A prospective study design would better determine the role of motor impairment in AD or aMCI subjects. Second, we did not find any consistent correlation between kinematics of AD and cognitive performance. This finding could be explained by a lack of statistical power due the small groups of subjects evaluated.

In summary, mild-to-moderate AD but not aMCI is significantly associated with abnormalities of motor function. These data support the issue that a pure memory impairment does not affect motor performance thus suggesting that motor abnormalities probably account for the compromising of other non-memory domains. Because of the prognostic value of motor impairment in AD subjects [32,46,50], our data may have relevant clinical and social implication. The slowing down of motor performance could indicate the worsening of cognitive functions and the early transition from aMCI to AD. However, longitudinal studies on larger samples of subjects are needed to confirm and extend our findings and to clarify the role of kinematic analysis as a useful tool for the screening of dementia.

## Acknowledgements

This study was supported by grants from MIUR, Sicilian Regional Government (progetto obiettivo P.S.R. ex art 54, l.r. 30/93, 2000–2002) to RC and Italian Ministero della Salute (ICS 120.3/RA00.84) to RC.
